# Flexor carpi radialis H-reflex in different body positions in patients with post-stroke

**DOI:** 10.3389/fneur.2022.1004822

**Published:** 2022-11-08

**Authors:** Jia-Yin Ma, Jia-Jia Wu, Mou-Xiong Zheng, Xu-Yun Hua, Chun-Lei Shan, Jian-Guang Xu

**Affiliations:** ^1^Center of Rehabilitation Medicine, Yueyang Hospital of Integrated Traditional Chinese and Western Medicine, Shanghai University of Traditional Chinese Medicine, Shanghai, China; ^2^Engineering Research Center of Traditional Chinese Medicine Intelligent Rehabilitation, Ministry of Education, Shanghai, China; ^3^Department of Traumatology and Orthopedics, Yueyang Hospital of Integrated Traditional Chinese and Western Medicine, Shanghai University of Traditional Chinese Medicine, Shanghai, China; ^4^School of Rehabilitation Science, Shanghai University of Traditional Chinese Medicine, Shanghai, China

**Keywords:** H-reflex, spinal stretch reflex, motor control, postural control, stroke

## Abstract

**Background:**

Spinal stretch reflex (SSR) hyperexcitability reflected by the H-reflex has been reported in more strongly affected extremities after stroke. The H-reflex in the lower extremities is modulated by body position normally and alternatively modulated post-stroke.

**Objective:**

This study aimed to preliminarily explore how upper extremity (UE) H-reflexes are modulated by body position after stroke, which remains unknown.

**Materials and methods:**

Three patients after stroke with hemiparesis/hemiplegia were included. Bilateral flexor carpi radialis (FCR) H-reflexes were examined in the supine position while standing. Other clinical evaluations include the modified Ashworth scale (MAS) and postural stability measurement.

**Results:**

The three cases herein showed that (1) SSR excitability was higher in more strongly affected UEs than less-affected UEs, (2) down-modulation of SSR excitability occurred in less-affected UEs in static standing compared with the supine position, but modulation of SSR excitability in more-affected UEs varied, and (3) bilateral UE SSR excitability in case 3 was down-modulated the most. Moreover, case 3 showed no difference in muscle tone of the more affected UE between supine and standing positions, and case 3 showed the best postural stability.

**Conclusion:**

Spinal stretch reflex hyperexcitability in strongly affected UEs could commonly occur in different phases of recovery after stroke. Down-modulation of SSR excitability could occur in less-affected UEs in the standing position compared with the supine position, while modulation of SSR excitability might be altered in strongly affected UEs and vary in different phases of recovery. There could be some correlation between postural control and UE SSR hyperexcitability. The H-reflex may help to offer a new perspective on rehabilitation evaluation and interventions to promote UE motor control after stroke.

## Introduction

Upper extremity (UE) movement dysfunction is the most common and stable symptom in stroke survivors (1), often presenting with increased flexion synergy patterns or spasticity and difficulty in voluntary isolated movements. Flexion synergy patterns are observed in patients with hemiplegia in different body positions (2) and during dynamic activities (3), which seriously affect their activities of daily life.

It is widely accepted that the increase in flexion synergy patterns is mediated by an exaggerated spinal stretch reflex (SSR). The H-reflex has been usually employed as a measure of SSR excitability to investigate spinal neuronal pathway health in patients with neurological disorders. It is evoked by low-intensity electrical stimulation of the Ia afferent, resulting in the monosynaptic excitation of motor neurons. Changes in H-reflex amplitude are usually used to assess different types of inhibition acting on Ia afferent terminals, including homosynaptic depression, presynaptic inhibition, reciprocal Ia inhibition, Ib inhibition, and recurrent inhibition (4).

It has been reported that SSR hyperexcitability is reflected by the H-reflex in more strongly affected upper and lower extremities after stroke (5, 6). Several studies found that the soleus H-reflex varies in different body positions and is specifically down-modulated when sitting or standing compared with the prone position in healthy participants (7, 8). One previous study has also shown that the soleus H-reflex was up-modulated on both sides when standing compared with the prone position in patients with spasticity after stroke (5). Collectively, most previous studies focused on the relationship between soleus H-reflex modulation and postural control in healthy adults and patients with upper motor neuron disease. Although it has been reported that increased flexion synergy patterns in more strongly affected UEs were significantly associated with impaired postural control after stroke (9), UE H-reflex characteristics in different body positions, and at different phases of stroke recovery remain unknown. Thus, we hypothesized that H-reflex modulation in UEs might vary in different body positions in patients after stroke, and modulation of SSR excitability in UEs might be correlated to postural control.

The objective of this study was to preliminarily explore how UE H-reflexes were modulated by different body positions after stroke. The study presented three cases in different phases of stroke recovery. The muscle chosen for the H-reflex examination was the flexor carpi radialis (FCR), which has been commonly used. Furthermore, measurements of the modified Ashworth scale (MAS) in two different body positions as well as postural stability in static standing were also assessed. We hope that this study can provide new perspectives for evaluation and intervention in the rehabilitation of strongly affected UEs after stroke.

## Patient perspective

The patients enrolled in this study had been participating actively in rehabilitation before the study commenced, and they had a great interest in learning about their motor recovery in their UE. Thus, they agreed to be enrolled in the study and were well informed regarding the study's purpose and their rights as participants.

## Case description

### Case 1

A 38-year-old right-hand-dominant male patient suddenly presented with slurred speech, a drooping mouth, and weakness in his left extremities. The patient was diagnosed with ischemia in the right parietal lobe and received thrombolytic therapy. Two hours later, he had a severe headache and was nauseated. A new CT scan showed a hemorrhage in his right basal ganglia. Craniotomy was suggested but refused by the patient. He was then given conservative treatment. After 18 days in stable condition, he was referred to rehabilitation to receive physical, occupational, speech, and acupuncture therapy. He was admitted to our study 59 days after stroke onset, in the rehabilitation phase of recovery. The patient's relevant past medical history included hypertension and long-term use of valsartan (80 mg qd) to manage blood pressure.

### Case 2

A 38-year-old right-hand-dominant male patient underwent cerebral hemorrhage in his left basal ganglia as well as radiographic coronal area and had a seizure. In the emergency room, the intracerebral hematoma was removed *via* the left lateral fissure under general anesthesia, and an intracranial pressure probe was placed. After 38 days in stable condition, the patient was referred to rehabilitation for physical, occupational, speech, and acupuncture therapy. He was admitted to our study 221 days after stroke onset, in the chronic phase of recovery.

Six and a half months after stroke onset, to reduce spasticity in his more affected UE, he received multipoint Botox injections in the right pectoralis major, biceps brachii, brachioradialis, teres pronator, and triceps surae.

### Case 3

A 71-year-old right-hand-dominant female patient presented with weakness in her left extremities and dizziness. She was diagnosed with ischemia in her right basal ganglia and lateral paraventricular region. There were also multiple older ischemic foci and lumens in the bilateral basal ganglia, lateral ventricle, and the centrum semiovale. The patient then received active symptomatic treatment. One day later, her motor dysfunction had gotten worse and she suffered from slurred speech and occasional choking. Ganglioside and other active symptomatic treatments were further prescribed. After 28 days in stable condition, she was referred to rehabilitation for physical, occupational, speech, and acupuncture therapy. The patient was admitted to our study 50 days after stroke onset, in the sub-acute phase of recovery.

## Materials and methods

The case data were retrieved from the Yueyang Hospital of Integrated Traditional Chinese and Western Medicine, Shanghai University of Traditional Chinese Medicine. We included three cases in total. The present study followed the international recommendations of the CARE checklist for drafting reports of case studies (10). All participants provided informed consent. The study included the following evaluation methods.

### Electrophysiological evaluation

The FCR H-reflexes recruitment was recorded using electrodiagnostic equipment (Dantec Keypoint 9033A; Natus Medical Incorporated, Middleton, WI, USA). The signals were amplified and digitized using a three-channel amplifier (Dantec Co., Ltd, Denmark). Data were collected at a sampling rate of 48 kHz and band-pass filtered (low-pass 2000 Hz, high-pass 20 Hz). Active shielding and grounding of the cables were used to minimize the power line (50 Hz) interference. Before FCR H-reflex examination, the skin of the patient's forearm was cleaned with 75% isopropyl alcohol solution, and FCRs in bilateral UEs were located by finding the fullest FCR muscle belly, at about one-third of the distance between the medial epicondyle and the radial styloid (11). Surface electromyograms (EMGs) were recorded from the FCR muscle using 20-mm Ag/AgCl square electrodes in a monopolar configuration. The recording electrode was attached to the FCR while the reference electrode was placed on the radial styloid. The ground electrode was placed on top of the clavicle on the same side as the tested FCR. The electrodes were attached while the patient was in the supine position and was kept in place for the examination in the standing position, ensuring the same recording locations. To recruit the FCR H-reflex, the median nerve was stimulated at the elbow using a bipolar stimulation electrode, which was immobilized by an armlet, and the stimulation site was marked with a pen to ensure the same stimulation site during the whole procedure. The optimal stimulation site of the median nerve was identified using the stimulation electrodes. A rectangular pulse of 1.0 ms duration was delivered to stimulate the median nerve. The interval between stimulations was 10 s.

With the intensity of the electrical stimulation current increasing gradually starting from 0.0 mA, the H-reflex trace began to appear in the EMG traces as the excitation threshold of the Ia afferents was reached. When increasing stimulus intensity, the M-wave appeared and was followed by the H-reflex as the excitation threshold of α motor neurons was reached. The peak-to-peak amplitude of the H-reflex increased until a maximum value (Hmax) was reached and subsequently decreased. As more α motor neurons were recruited, the M-wave increased until its maximum value (Mmax) was reached. Hmax and Mmax were recorded, and the Hmax/Mmax ratio was calculated, which was considered to represent the proportion of the motor neurons recruited by a monosynaptic reflex in the motor neuron pool and reflect the excitability of motor neurons and inhibition that mediate the Ia afferent volley (4). In order to avoid potential systemic changes affecting all parameters in the trials, Mmax was recorded under the respective testing conditions as a reference.

**Figure 1:**
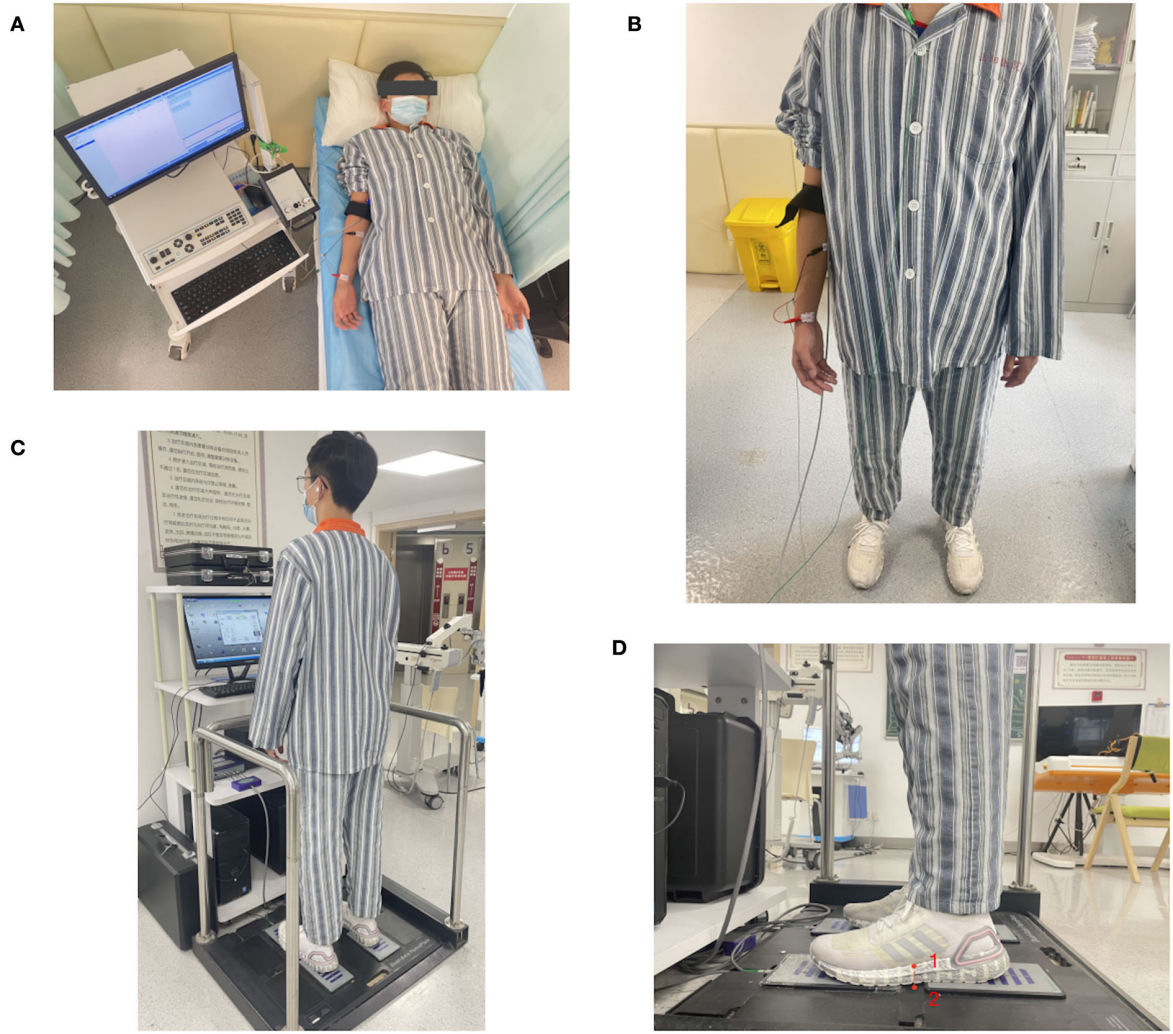
Flexor carpi radialis (FCR) H-reflex examination in the **(A)** supine and **(B)** static standing positions; **(C)** When evaluating postural stability in static standing without support over 5 s, participants were instructed to stand on the force plates with their feet hip-width apart, head and trunk centered and neutral, and bilateral UEs naturally hanging; **(D)** Each foot of the patient was across the gap of 2 the force plates on one side, and the midpoint of the medial foot arch (red point 1) was just above the midpoint of the gap (red point 2) when the patient stood on the force plates.

The FCR H-reflex was examined in bilateral UEs in both the supine and standing positions. First, the patients were instructed to lie in the supine position on the examination bench, keeping their extremities extended and relaxed, with both palms facing up, the legs slightly spread, and the head and trunk in a centered neutral position. The patients were instructed to relax on the couch for 2 min before the FCR H-reflex examination. After the FCR H-reflex examination in the supine position, the patients were instructed to change the standing position without support. In standing position, the patients were asked to stand still with their head and trunk centered and neutral, their feet hip-width apart with their toes facing forward, and UEs hanging naturally on both sides of the torso. The examination in the standing position was started after a 2-min relaxation period. The examination is illustrated in Figure 1.

### Muscle tone evaluation of the more-affected upper extremities

The MAS was assessed in the elbow and wrist flexors of the more strongly affected UEs in both the supine and static standing positions. The proximal part of the UE was stabilized, and the distal part was moved passively by the evaluator through its available range of motion from a position of maximal flexion to maximal extension over a period of about 1 s by counting “one thousand and one”. Two repetitions were performed, and the evaluator took an average score of the two.

The resistance of muscle tone was scored on the following 6-point scale: 0, no increased resistance; 1, slightly increased resistance (catch followed by relaxation or minimal resistance at the end of the range of motion); 1+, slightly increased resistance (catch followed by minimal resistance throughout less than half of the range of motion); 2, clear resistance throughout most of the range of motion; 3, strong resistance with which passive movement is difficult; and 4, rigid flexion or extension.

### Postural stability in static standing

The patients were instructed to stand with their head and trunk upright, eyes looking forward, both UEs hanging naturally, and their feet hip-width apart standing on force plates (E-LINK FP3 Force Plates System; Biometrics Ltd., Ynysddu, UK). The patients were instructed to stand still on the force plates without any assistance for 5 s. The evaluation is illustrated in Figure 1.

The force plates recorded their center of pressure (COP) trajectories over the 5 s of standing. To evaluate the postural stability, mean sway amplitude (MSA, root mean square of the COP trajectories) was automatically calculated in the system and was returned as a percentage, referring to the rate of MSA, with a lower percentage indicating better postural stability.

## Results

### Electrophysiological evaluation

**Figure 2:**
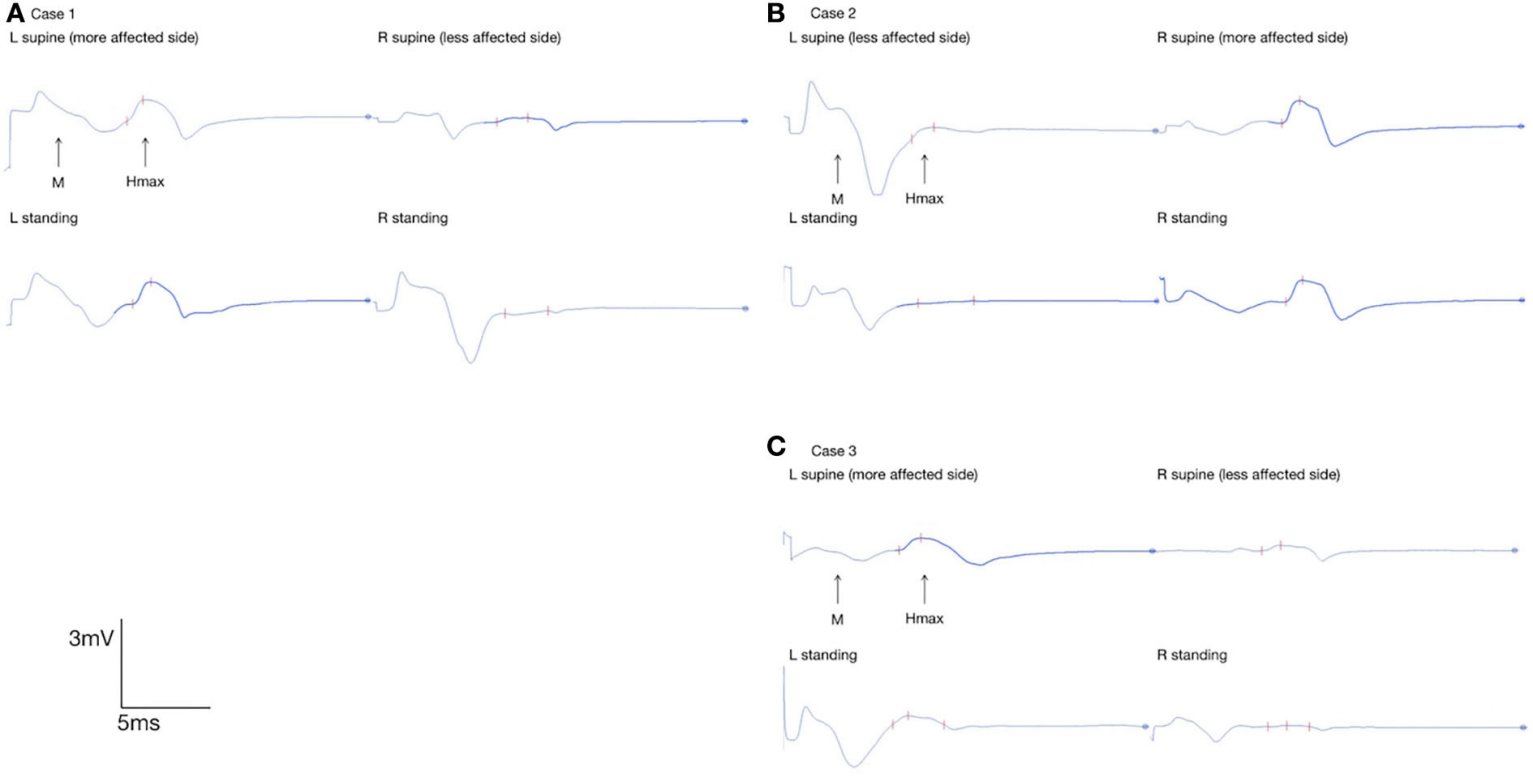
Flexor carpi radialis H-reflex in bilateral UEs in **(A)** case 1, **(B)** 2, and **(C)** 3; FCR Hmax of the more-affected UE and less-affected UE in the supine position and static standing were marked with red marks on the EMG interface.

Flexor carpi radialis (FCR) H-reflex data collected in the three cases are presented in Table 1 and Figure 2. In Table 1, the difference in FCR Hmax/Mmax ratios between the supine and standing positions is presented. This variation was calculated as follows:


$$\begin{array}{l} variation\;(\%)\\ \;=\;\frac{Hmax/Mmax\;in\;standing-Hmax/Mmax\;in\;supine}{Hmax/Mmax\;in\;supine}\\ \;\times 100\% \end{array}$$

Table 1Clinical evaluation of cases.

**Table:** 

**Hmax/Mmax ratio of bilateral UEs**						
	**Supine**		**Standing**		**Variation (%)**	
	**More affected**	**Less affected**	**More affected**	**Less affected**	**More affected**	**Less affected**
Case 1	0.39	0.08	0.41	0.07	+5.12	−12.50
Case 2	0.53	0.22	0.51	0.05	−3.77	−77.27
Case 3	0.32	0.13	0.21	0.03	−34.38	−76.92

**Table:** 

**Modified Ashworth scale of more-affected UE flexors**				
	**Supine**		**Standing**	
	**Elbow**	**Wrist**	**Elbow**	**Wrist**
Case 1	2	1+	3	2
Case 2	2	2	3	3
Case 3	1	1	1	1

**Table:** 

**Mean sway amplitude of center of pressure**		
	**ML axis**	**AP axis**
Case 1	5.10%	5.30%
Case 2	2.30%	7.50%
Case 3	0.90%	3.20%

Variation—the difference of Hmax/Mmax ratios between supine and standing; ML axis, mediolateral axis; AP axis, anteroposterior axis.

Despite the body position, the FCR Hmax/Mmax ratios of the more strongly affected UEs were higher than those of the less-affected UEs. Between the supine and standing positions, the FCR Hmax/Mmax ratios of the less-affected UE decreased by 12.50% in case 1, 77.27% in case 2, and 76.92% in case 3. However, the FCR Hmax/Mmax ratios of the more strongly affected UE increased by 5.12% in case 1, decreased by 3.77% in case 2, and decreased by 34.38% in case 3.

### Muscle tone evaluation of the more-affected upper extremity

Modified Ashworth scale (MAS) data in the three cases are presented in Table 1. Between the supine and standing positions, the MAS score increased from 1+ (wrist) and 2 (elbow) to 2 (wrist) and 3 (elbow) in case 1 and from 2 (wrist) and 2 (elbow) to 3 (wrist) and 3 (elbow) in case 2. However, in case 3, the MAS scores of the more strongly affected wrist and elbow were both 1 and did not vary between the supine and standing positions.

### Postural stability in static standing

**Figure 3:**
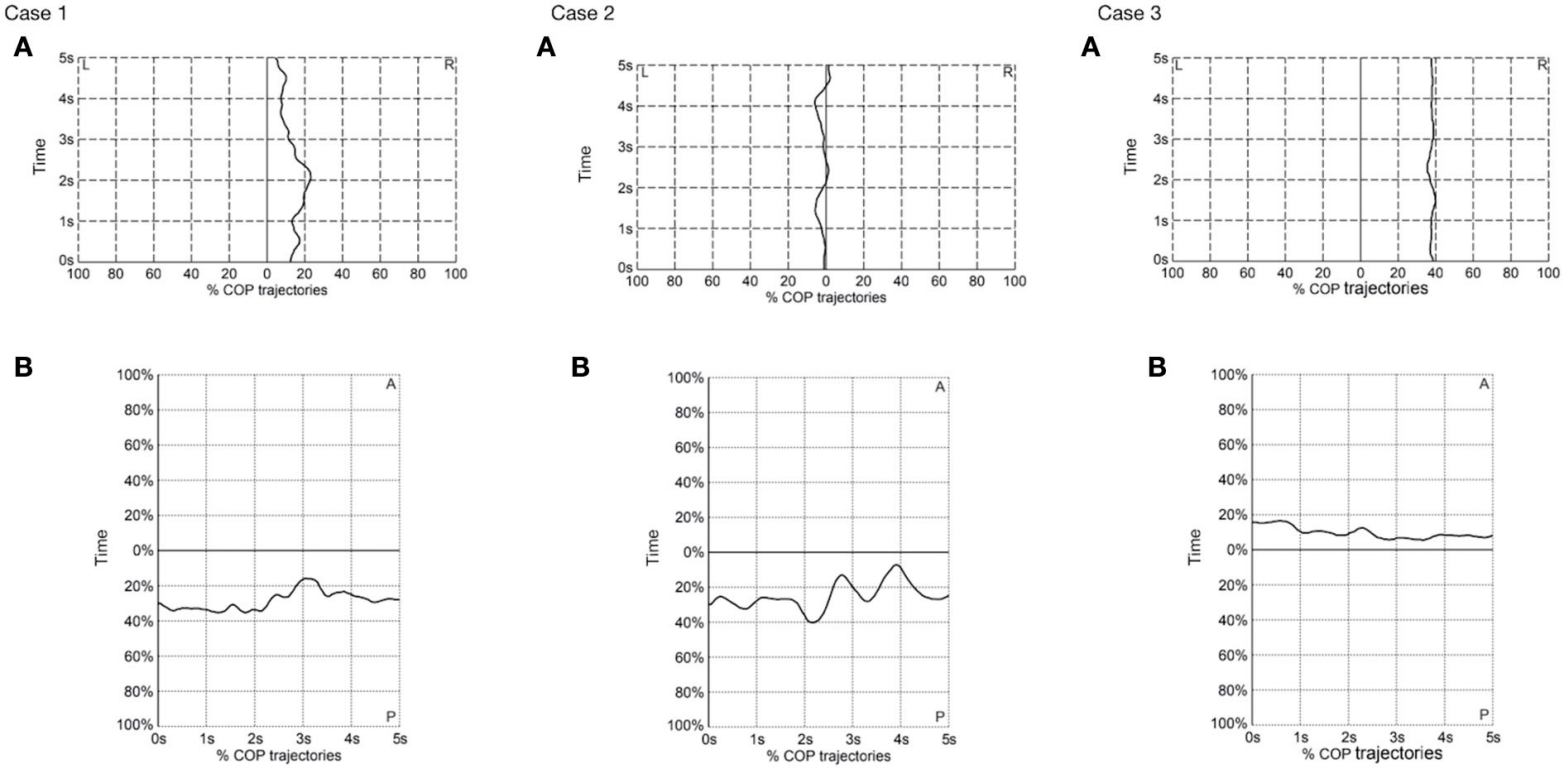
Center of pressure trajectories in **(A)** mediolateral (ML) and **(B)** anteroposterior (AP) axes in the three cases in the static standing position. L, left; R, right; COP, center of pressure.

The postural stability was assessed in both mediolateral and anteroposterior axes (Table 1 and Figure 3). No joint stiffness was seen in the three cases. Case 3 had the lowest MSA of COP in both axes in the static standing, demonstrating the best postural stability among the three cases.

## Discussion

### FCR H-reflex of more-affected and less-affected UEs

Flexor carpi radialis (FCR) H-reflex measurements showed that the Hmax/Mmax ratios of the more-affected UEs were higher than those of the less-affected UEs in both the supine and standing positions in all three cases, which is consistent with a previous study in patients with chronic stroke (6), indicating more SSR excitability in more-affected UEs. To be noted, case 3 was in the sub-acute phase and case 1 was in the rehabilitation phase. Thus, the underlying neural mechanisms could be similar in these different phases of recovery. Imbalanced supraspinal descending regulation and abnormal intraspinal processing have been considered the causes of SSR hyperexcitability (12). The reticulospinal tract (RST), as supraspinal descending projection, has been confirmed to regulate the intraspinal motor network, having a key effect on regulating SSR (13). The RST output is different in the sub-acute and chronic stages after stroke (14, 15). It remains unclear what SSR excitability in bilateral UEs might be like in different phases of recovery and whether UE SSR excitability could be altered by changes in the RST after stroke.

### FCR H-reflex in the supine position and static standing

Previous research has shown obvious down-modulation of lower extremity (LE) SSR excitability in the sitting and standing positions than in the prone position in healthy subjects (5). In patients with chronic stroke, the SSR excitability in bilateral LEs was generally up-modulated when compared with healthy controls (7). In the current study, the results showed that the Hmax/Mmax ratios of the less-affected UEs were lower in the standing position than in the supine position. However, it remains unknown and calls for further study, on whether modulation of the SSR excitability in less-affected UEs is different from that in healthy controls. Moreover, Hmax/Mmax ratios in the supine and standing positions in the more-affected UEs varied among the three cases. In general, Hmax/Mmax ratios in cases 1 and 2 varied little in the more-affected UEs between the two body positions, while that of case 3 was lower in static standing. Modulation of the SSR excitability in different body positions might vary in different phases of recovery, which should be further studied.

Spasticity can be divided into its neurological and biomechanical components (16). The neurological components relate to central motor lesions, which cause supraspinal drive and defective processing of afferent signals with impaired short- and long-latency reflexes (16). The biomechanical components relate to secondary changes in mechanical muscle fiber, collagen, and tendons, resulting in spastic muscle tone (16). For case 3, in the sub-acute stage of stroke recovery, we inferred that the patient's neurological components were more dominant in her spasticity (or flexion synergy patterns) in the more-affected UE.

The UEs play an important role not only in motor control but also in balance and postural control. A previous study confirmed that increased flexor muscle tone or flexion synergy patterns when standing have been regarded as a compensation strategy for postural instability (2). Looking at the present postural stability and MAS measurements, increased flexor spasticity when standing in cases 1 and 2 might be compensating for postural instability more than in case 3. When looking at the results of postural stability measurements with FCR H-reflex modulation, the FCR H-reflex excitability of the more-affected UE in case 3 was the lowest among the UEs and cases. Additionally, FCR H-reflex excitability in the more-affected UE in case 3 was down-modulated between the supine position and static standing. Referring to the difference in modulation in LEs between healthy subjects and patients with chronic stroke, SSR excitability in the more-affected UE tended to be closer to healthy patterns in case 3. Additionally, case 3 showed the best postural stability in standing among, and we hypothesized that there could be a correlation between postural control and SSR excitability of the more-affected UE.

In addition to the fact that the RST has a strong role in modulating SSR excitability, it also plays a key role in both postural control and motor control involving proximal and distal UE muscles (17). It has been confirmed that RST output is altered in the sub-acute and chronic phase of stroke recovery and is significantly related to flexion synergy patterns in the more-affected UEs of patients with chronic stroke (18). It should be further studied whether changes in RST output affect UE SSR excitability and underlie the correlation mentioned above.

### Limitations

The present study had some advantages and limitations. This study was the first to measure FCR H-reflexes in UEs in patients after stroke in different body positions, and the first to preliminarily explore the possible relationships between postural control and UE SSR excitability after stroke. However, this study included only three cases. Thus, the results were drawn from limited sample size and obtained after data collection at a single time point. Further studies using larger sample sizes and long-term follow-up should be conducted to obtain more comprehensive results. Variables (e.g., age, recovery phase, area of brain lesion, etc.) and factors (e.g., the experiment order of body positions) should be also controlled in further study. Some devices or methods are also required to ensure the consistency of body positions among the participants. Additionally, more functional situations (e.g., standing with eyes open or closed, standing on the ramp, walking, etc.) could be considered. Moreover, more evaluation methods such as brain functional imaging and evoked potentials could be applied to get more holistic results and to further explore the possible mechanisms that underlie postural control and motor control deficits after stroke.

## Conclusion

Spinal stretch reflex (SSR) hyperexcitability in strongly affected UEs could commonly occur in different phases of recovery after stroke. Down-modulation of SSR excitability could occur in less-affected UEs in the standing position compared with the supine position, while modulation of SSR excitability might be altered in strongly affected UEs and vary in different phases of recovery. There could be some correlation between postural control and UE SSR hyperexcitability. We further hypothesized that changes in RST output might underly SSR hyperexcitability in different phases of stroke recovery and altered SSR modulation in different body positions. We hope that the H-reflex can help to offer a new perspective for evaluation and intervention in UE rehabilitation after stroke.

## Data availability statement

The original contributions presented in the study are included in the article/supplementary material, further inquiries can be directed to the corresponding authors.

## Ethics statement

The studies involving human participants were reviewed and approved by the Ethics Committee of Yueyang Hospital, affiliated with Shanghai University of Traditional Chinese Medicine. The patients/participants provided their written informed consent to participate in this study. Written informed consent was obtained from the individual(s) for the publication of any potentially identifiable images or data included in this article.

## Author contributions

J-GX and C-LS designed the study. M-XZ and X-YH interpreted the results. J-YM and J-JW analyzed the data and wrote the manuscript. All authors revised and approved the final version of the manuscript.

## Funding

This study was supported by the National Key R&D Program of China (Grant Nos. 2018YFC2001600 and 2018YFC2001604), the National Natural Science Foundation of China (Grant Nos. 81802249, 81871836, and 81902301), the Shanghai Rising-Star Program (Grant No. 19QA1409000), and the Shanghai University of Traditional Chinese Medicine (Grant No. 2021yyjq07).

## Conflict of interest

The authors declare that the research was conducted in the absence of any commercial or financial relationships that could be construed as a potential conflict of interest.

## Publisher's note

All claims expressed in this article are solely those of the authors and do not necessarily represent those of their affiliated organizations, or those of the publisher, the editors and the reviewers. Any product that may be evaluated in this article, or claim that may be made by its manufacturer, is not guaranteed or endorsed by the publisher.
